# Construction and Characterization of Normalized cDNA Libraries by 454 Pyrosequencing and Estimation of DNA Methylation Levels in Three Distantly Related Termite Species

**DOI:** 10.1371/journal.pone.0076678

**Published:** 2013-09-30

**Authors:** Yoshinobu Hayashi, Shuji Shigenobu, Dai Watanabe, Kouhei Toga, Ryota Saiki, Keisuke Shimada, Thomas Bourguignon, Nathan Lo, Masaru Hojo, Kiyoto Maekawa, Toru Miura

**Affiliations:** 1 Graduate School of Environmental Science, Hokkaido University, Sapporo, Japan; 2 School of Biological Sciences, The University of Sydney, Sydney, New South Wales, Australia; 3 NIBB Core Research Facilities, National Institute for Basic Biology, National Institutes of Natural Sciences, Okazaki, Japan; 4 Department of Basic Biology, School of Life Science, Graduate University for Advanced Studies, Okazaki, Japan; 5 Graduate School of Science and Engineering, University of Toyama, Toyama, Japan; 6 Graduate School of Bioagricultural Sciences, Nagoya University, Nagoya, Japan; 7 Ishikawa Museum of Natural History, Kanazawa, Japan; 8 Department of Biological Sciences, National University of Singapore, Singapore, Singapore; 9 Tropical Biosphere Research Center, University of the Ryukyus, Okinawa, Japan; Queen's University Belfast, United Kingdom

## Abstract

In termites, division of labor among castes, categories of individuals that perform specialized tasks, increases colony-level productivity and is the key to their ecological success. Although molecular studies on caste polymorphism have been performed in termites, we are far from a comprehensive understanding of the molecular basis of this phenomenon. To facilitate future molecular studies, we aimed to construct expressed sequence tag (EST) libraries covering wide ranges of gene repertoires in three representative termite species, *Hodotermopsis* *sjostedti* , *Reticulitermessperatus* and *Nasutitermestakasagoensis* . We generated normalized cDNA libraries from whole bodies, except for guts containing microbes, of almost all castes, sexes and developmental stages and sequenced them with the 454 GS FLX titanium system. We obtained >1.2 million quality-filtered reads yielding >400 million bases for each of the three species. Isotigs, which are analogous to individual transcripts, and singletons were produced by assembling the reads and annotated using public databases. Genes related to juvenile hormone, which plays crucial roles in caste differentiation of termites, were identified from the EST libraries by BLAST search. To explore the potential for DNA methylation, which plays an important role in caste differentiation of honeybees, tBLASTn searches for DNA methyltransferases (*dnmt1, dnmt*2 and *dnmt*3) and methyl-CpG binding domain (mbd) were performed against the EST libraries. All four of these genes were found in the *H* *. sjostedti* library, while all except *dnmt*3 were found in *R* *. speratus* and *N* *. takasagoensis* . The ratio of the observed to the expected CpG content (CpG O/E), which is a proxy for DNA methylation level, was calculated for the coding sequences predicted from the isotigs and singletons. In all of the three species, the majority of coding sequences showed depletion of CpG O/E (less than 1), and the distributions of CpG O/E were bimodal, suggesting the presence of DNA methylation.

## Introduction

Termites, one of the major social insect groups, live in colonies and construct complex societies with highly sophisticated division of labor among castes, which show distinctive behavior and morphology for their specialized tasks [1,2]. Division of labor is the key to efficient colony performance, leading to the ecological success of termites particularly in tropical and subtropical terrestrial regions [2].

Distinct castes express different sets of genes and are differentiated from each other through differential gene expression during postembryonic development in response to environmental factors [3,4]. To date, a significant number of studies that focused on differential gene expression among castes and among individuals in the course of caste differentiation have been reported in termites (reviewed in Miura & Scharf [4]). These studies successfully identified genes with caste-specific or -biased expressions, and up- or down-regulated genes during caste differentiation. However, only a few castes in a few species have been investigated. Termites are represented by more than 2600 extant species [5] and exhibit considerable diversity in morphology and behavior of castes and caste developmental pathways [6]. At present, a comprehensive understanding of caste polymorphism of termites from the molecular viewpoint is still far from being reached. To facilitate gene expression analyses in termites, it is important to construct expressed sequence tag (EST) libraries, which are used as an information source for gene discovery, gene structure identification, and so on [7-10].

To date, large-scale EST libraries have been constructed in only a handful of termite species. In *Coptotermes* *formosanus* (Rhinotermitidae), Husseneder et al. [11] obtained 4,726 ESTs generated from an assembly of 7,663 sequence reads by Sanger sequencing of a cDNA library originating from various castes with normalization of cDNA (which equalizes transcript concentrations in a cDNA library) [12,13]. Zhang et al. [14] generated normalized cDNA library from workers, soldiers, nymphs, and male and female alates of *C* *. formosanus* , and obtained 16,691 contigs and 9,248 singletons that resulted from assembly of 131,636 Sanger sequence reads. In *Reticulitermes* *flavipes* , a total of 15,259 Sanger reads were obtained from non-normalized cDNA generated from alates, workers, soldiers, and larvae by Steller et al. [15]. Recently Hojo et al. [16] performed 454 pyrosequencing for transcriptomes of frontal gland tissues to identify genes involved in the synthesis of terpenoid defensive secretion in *Nasutitermestakasagoensis* (Termitidae) and generated 1,189 contigs that were assembled from 50,290 clean reads. Huang et al. [17] used heads of workers of *Odontotermes* *formosanus* for Illumina sequencing and generated 57 million reads that were assembled into 221,728 contigs. In addition to those large-scale EST sequencing, a number of smaller scale EST projects were carried out in termites. As of June 2013, 164,150 ESTs from termite species were deposited in the dbESTs of NCBI GenBank (http://www.ncbi.nlm.nih.gov/dbEST/dbEST_summary.html) and 3 projects that produced 526,778 sequences in total in the SRA of GenBank, including a metagenomic analysis that primarily focused on gut symbionts of termites. However, the EST libraries generated to date are likely to be missing significant parts of the termite transcriptome gene repertoire. cDNA library construction using many castes, as well as massive sequencing with next generation technologies, are both required to obtain a wide range of the gene repertoire. To date, no study has combined both of these aspects.

In this study we generated normalized cDNA libraries from RNA pools of whole bodies (excluding the digestive tracts) of almost all castes, sexes and developmental stages in three species of termites, *Hodotermopsis* *sjostedti* (Termopsidae), *R* *. speratus* (Rhinotermitidae) and *N* *. takasagoensis* (Termitidae). We sequenced each of the libraries using 454 pyrosequencing technology (GS FLX Titanium System). The three termite species belong to different families that are phylogenetically distant from one another; *H* *. sjostedti* is at relatively basal position, *R* *. speratus* is in an intermediate position, and *N* *. takasagoensis* is at the most apical part in the termite phylogenetic tree [18]. Moreover, their caste developmental pathways markedly differ (Figure S1): *H* *. sjostedti* has a linear developmental pathway, lacking a true worker caste, while *R* *. speratus* and *N* *. takasagoensis* have a bifurcated developmental pathway with a true worker caste. Morphology of the castes also differs, especially among soldiers; soldiers of *H* *. sjostedti* and *R* *. speratus* have elongated mandibles for biting enemies, while those of *N* *. takasagoensis* have reduced mandibles and enlarged frontal glands which they use to spray chemicals against enemies. Importantly, manipulation experiments for studying caste differentiation, such as hormone application and RNA interference techniques, have been established in these species (for example, Ogino et al. [19] and Hattori et al. [20] for *H* *. sjostedti* , Tsuchiya et al. [21] and Nambu et al. [22] for *R* *. speratus* , and Toga et al. [23] for *N* *. takasagoensis* ). With these techniques, molecular and physiological studies on caste differentiation have been performed and have provided a significant amount of knowledge on the molecular basis of caste polymorphism (reviewed in Miura & Scharf [4]). Construction of large-scale EST databases for these species will allow us to carry out gene expression analyses of caste polymorphism more efficiently.

In addition to EST library construction, we explored the possibility of DNA methylation in the three termite species by examining whether gene sequences of DNA (cytosine-5) methyltransferases, namely, *dnmt1*, *dnmt2* and *dnmt3*, and methyl-CpG binding domain, *mbd*, were present in the EST libraries. Furthermore, we estimated DNA methylation levels of coding sequences by computational analysis on the EST data. DNA methylation is known to be one of the most important mechanisms for generating differential gene expression triggered by environmental cues [24]. In animals, DNA methylation mostly occurs in cytosine nucleotides that are located next to guanine nucleotides, which are known as CpG dinucleotides [25,26]. In invertebrates, exon regions are the main targets of DNA methylation [27,28]. Furthermore, it is known that DNA methylation can be predicted from normalized CpG nucleotide content, CpG O/E, which is the ratio of observed and expected frequencies of CpG sequences [26]. Due to hyper mutability of methylated cytosines to thymines [29,30], CpG O/E of heavily methylated genes in germlines decreases over evolutionary time [26,30,31]. Here, we calculated CpG O/E of coding sequences predicted from the EST data and discussed DNA methylation levels of the three termite species.

## Results and Discussion

### Normalized cDNA library construction

The primary purpose of this study is to construct EST libraries that contain wide ranges of expressed-gene repertoires and thus we collected as many castes, sexes, and developmental stages of termite individuals as possible, because the different types of individuals may express different sets of genes [4]. We extracted total RNA from 18, 26, and 25 categories of individuals that were classified based on caste/sex/developmental stage in *Hodotermopsis* *sjostedti* , *Reticulitermessperatus* , and *Nasutitermestakasagoensis* , respectively (Tables S1-S3). These RNA transcripts from different categories were pooled for each of the species. To facilitate the sequencing of rare transcripts, we applied a normalization technique to the cDNA generated by reverse transcription of the RNA.

### Sequencing and assembly of normalized cDNA libraries

The normalized cDNA libraries were sequenced with a 454 GS FLX Titanium system (Roche, Indianapolis, IN, USA). All of the reads obtained have been deposited in the DDBJ Sequence Read Archive (DRA) database under accession numbers DRA000538 and DRA001044 for *H* *. sjostedti* , DRA001045 for *R* *. speratus* , and DRA001046 for *N* *. takasagoensis* . After removing adaptor sequences and low quality nucleotides, 1.22 M, 1.32 M, and 1.39 M reads in *H* *. sjostedti* , *R* *. speratus* , and *N* *. takasagoensis* , respectively, were retained for further analyses, yielding a total of 401.8 M, 443.0 M, and 517.6 M bases (Table 1).

**Table 1:** Sequencing and assembly statistics in three termite species.

	*** Hodotermopsis sjostedti ***	*** Reticulitermes speratus ***	*** Nasutitermes takasagoensis ***
No. of reads	1,221,634	1,317,986	1,387,437
No. of bases	402,779,218	444,211,498	518,789,889
No. of clean reads	1,221,416	1,317,777	1,387,263
No. of clean bases	401,761,320	443,013,802	517,610,329
No. of isogroups	41,306	43,201	16,635
No. of isotigs	50,009	55,636	27,559
mean length of isotigs	621.5	654.6	820.0
median length of isotigs	494	506	681
N50 length of isotigs	711	773	974
No. of singletons	83,549	87,191	128,438
mean length of singletons	155.1	184.6	304.3
median length of singletons	71	80	346
N50 length of singletons	339	359	411
No. of singletons ≥ 100 bp	29,542	40,126	103,123
mean length of singletons ≥ 100 bp	318.1	321.7	358.6
median length of singletons ≥ 100 bp	337	338	381
N50 length of singletons ≥ 100 bp	399	388	411

To assemble the cleaned reads, we used a GS de novo Assembler (Newbler) version 2.5.3 (Roche) with cDNA mode. The assembling produced 49,919 isotigs, 90 contigs and 83,549 singletons in *H* *. sjostedti* , 55,476 isotigs, 160 contigs and 87,191 singletons in *R* *. speratus* , and 27,408 isotigs, 151 contigs and 128,438 singletons in *N* *. takasagoensis* . For simplicity, isotigs and contigs are hereafter collectively referred to as isotigs (n = 50,009 in *H* *. sjostedti* ; 55,636 in *R* *. speratus* ; and 27,559 in *N* *. takasagoensis* ; Table 1). Singletons less than 100 bp (n = 54,007 in *H* *. sjostedti* ; 47,065 in *R* *. speratus* ; 25,315 in *N* *. takasagoensis* ) were discarded for subsequent analyses.

### Transcriptome completeness

To assess transcriptome completeness of the EST libraries, we surveyed how many genes out of gene sets conserved among taxa are present in the EST libraries. We performed BLASTX searches for the isotigs and singletons with various E-value thresholds (≤1e-5, ≤1e-10, ≤1e-15 and ≤1e-20) against Core Eukaryotic Genes (CEGs), which consist of 458 conserved genes [32]. The BLASTX results indicated that most of the CEGs (98.3%, 98.3%, and 99.1% in *H* *. sjostedti* , *R* *. speratus* and *N* *. takasagoensis* respectively; Table 2) showed significant similarity to at least one of the isotigs and singletons even with the most stringent E-value threshold, 1e-20.

**Table 2:** Gene coverage of EST libraries of three termite species.

**Gene set**	*** Hodotermopsis sjostedti ***				*** Reticulitermes speratus ***				*** Nasutitermes takasagoensis ***			
	**1e-5**	**1e-10**	**1e-15**	**1e-20**	**1e-5**	**1e-10**	**1e-15**	**1e-20**	**1e-5**	**1e-10**	**1e-15**	**1e-20**
Core Eukaryotic Genes [458]*	458 (100.0)	457 (99.8)	455 (99.3)	450 (98.3)	457 (99.8)	455 (99.3)	453 (98.9)	450 (98.3)	458 (100.0)	457 (99.8)	456 (99.6)	454 (99.1)
orthologs present in all of the insect species [278]*	272 (97.8)	270 (97.1)	267 (96.0)	262 (94.2)	275 (98.9)	274 (98.6)	273 (98.2)	268 (96.4)	276 (99.3)	272 (97.8)	269 (96.8)	265 (95.3)
orthologs present in all but one of the insect species [1332]*	1312 (98.5)	1287 (96.6)	1259 (94.5)	1245 (93.5)	1319 (99.0)	1299 (97.5)	1278 (95.9)	1258 (94.4)	1315 (98.7)	1298 (97.4)	1273 (95.6)	1259 (94.5)
orthologs present in > 90% of the insect species [4969]*	4665 (93.9)	4569 (92.0)	4475 (90.1)	4392 (88.4)	4737 (95.3)	4652 (93.6)	4576 (92.1)	4473 (90.0)	4714 (94.9)	4621 (93.0)	4530 (91.2)	4438 (89.3)

Numbers (percentages) of the Core Eukaryotic Genes and OrthoDB6 orthologous gene groups across insects to which ESTs of termites showed BLASTX hit with E-values ≤1e-5, ≤1e-10, ≤1e-15, and ≤1e-20 are shown.

* The number in brackets indicates the total number of the Core Eukaryotic Genes or the orthologous gene groups obtained from OrthoDB6 database.

Furthermore, transcriptome completeness was assessed also by using gene sets conserved among insects. OrthoDB6 database defined orthologous gene groups among taxa [33] (http://cegg.unige.ch/orthodb6). We retrieved protein sequences of the orthologs that were conserved in all, all but one, and ≥90% of the insect species registered in OrthoDB6 (278, 1,332 and 4,969 gene groups respectively). Then BLASTX searches were performed for the isotigs and singletons against the OrthoDB6 gene sets. The BLASTX searches revealed that more than 94%, 93% and 88% of genes conserved among all, among all but one and among ≥90% of the insect species, respectively, were detected from the EST libraries with a 1e-20 E-value threshold (Table 2).

These results suggest that the EST libraries cover most of the genes conserved among broad ranges of taxa.

### Functional annotation

For functional annotation, the isotigs and singletons were subjected to BLASTX searches against the non-redundant (nr) database of GenBank with threshold values of ≤1e-4 for E-value and ≥40 for bit score. We found that 29,249 (36.8%) [24,003 (48.0%) and 5,246 (17.8%) of the isotigs and the singletons, respectively] in *H* *. sjostedti* , 32,191 (33.6%) [25,982 (46.7%) and 6209 (15.5%)] in *R* *. speratus* , and 42,815 (32.8%) [17585 (63.8%) and 25,230 (24.5%)] in *N* *. takasagoensis* showed similarity to at least one gene from the nr database based on the threshold. The lack of BLASTX hits for the majority of the isotigs and singletons may be due to a small number of deposited protein sequences of termites and closely related insects, i.e., cockroaches and mantises.

**Figure 1:**
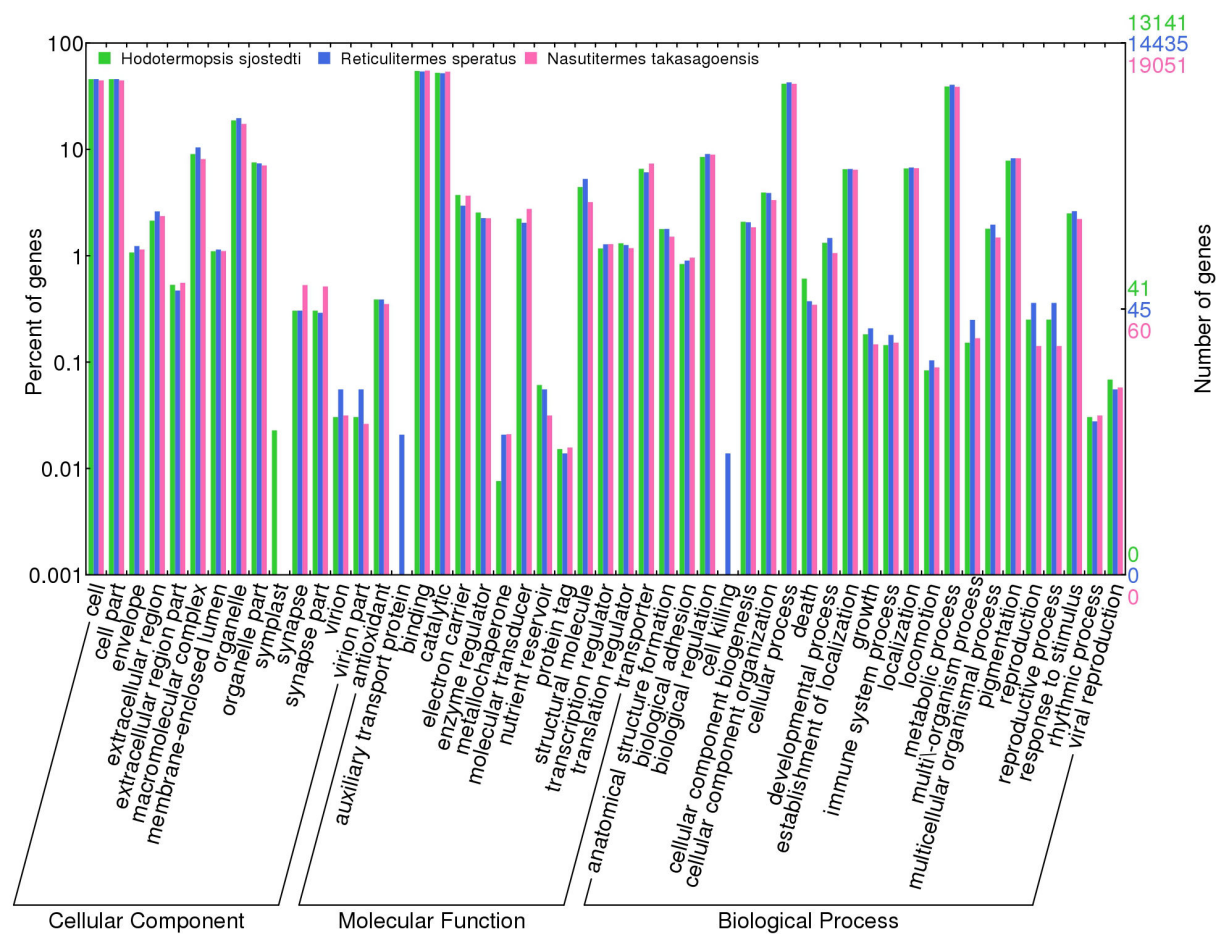
Gene Ontology annotation of EST libraries of three termite species. Frequency and percentage of the isotigs and singletons annotated by Gene Ontology terms are shown.

Based on the top hits of BLASTX searches, we assigned Gene Ontology (GO) annotation by using ID mapping data of the UniProt database. Of the total isotigs and singletons, 13,141 (16.5%) [11,774 (23.5%) and 1367 (4.6%) of the isotigs and the singletons, respectively], 14,435 (15.1%) [11664 (21.0%) and 2771 (6.9%)], and 19,051 (14.6%) [7942 (28.8%), 11109 (10.8%)] were annotated with GO terms in *H* *. sjostedti* , *R* *. speratus* and *N* *. takasagoensis* , respectively. Frequency of genes categorized into each of GO terms is shown in Figure 1.

We used the InterProScan software [34] with the Pfam database to search for functional protein domains in the isotigs and the singletons. In all of the termite species, the three most frequent domains were ‘Zinc-finger double domain’ (PF13465), ‘WD domain, G-beta repeat’ (PF00400), and ‘Zinc finger, C2H2 type’ (PF00096), with frequencies of 955, 471, and 234 respectively in *H* *. sjostedti* , 955, 471, and 234 in *R* *. speratus* , and 1303, 440 and 318 in *N* *. takasagoensis* (Table S4). This analysis also revealed that among the isotigs and singletons with no hits in the BLASTX searches, 136, 168, and 200 were predicted to contain functional protein domains.

### Identification of juvenile hormone-related genes

Juvenile hormone (JH) plays crucial roles in caste differentiation in termites [35,36]. The EST libraries were searched for JH-related genes that are listed in Table 3 using the TBLASTN algorithm with E-value threshold of 1e-20. As query sequences of the TBLASTN searches, we used insect orthologs of JH-related genes that were listed in Table 3 of The International Aphid Genomics Consortium [37]. We detected all of the JH-related genes from the EST libraries, except for ‘allatostatin receptor’ in the three termite species and ‘methoprene-tolerant’ in *H* *. sjostedti* . This information will be useful for future molecular studies on caste differentiation.

### Species distribution of BLASTX top hit genes

We examined species distributions of the top-hit genes in the BLASTX analysis with the nr database. In all of the termite species, *Tribolium* *castaneum* was the most frequent species in the distributions [2788 (11.7%), 2894 (11.8%), and 4256 (11.8%) of total isotigs and singletons with BLASTX hits in *H* *. sjostedti* , *R* *. speratus* , and *N* *. takasagoensis* , respectively; Table S5], followed by *Pediculus* *humanus* [2562 (10.7%), 2645 (10.8%), and 3935 (10.9%)]. The protist *Trichomonas vaginalis* was also present in the distributions but with much fewer hits [652 (2.7%), 251 (1.0%), 23 (0.06%)]. This is likely to result from contamination of RNA of symbiotic intestinal protists into the termite RNA samples, despite the fact that we removed guts of termites before RNA extraction. It is well known that lower termites such as *H* *. sjostedti* and *R* *. speratus* harbor symbiotic protists belonging to the order Trichomonadida [38,39], and the presence of symbiotic protists has also been suggested in *Nasutitermes* [40]. By summarizing counts of the species distribution, we found that more than 80% of blast top hits (81.3%, 81.9% and 84.1%) were to insects and other arthropods, and 5% or less were to protists and bacteria (protists: 3.7%, 2.3% and 1.0%; bacteria: 1.3%, 1.3% and 0.84%). Compared to an EST library of *Coptotermes* *formosanus* in which 42% of the top BLAST hits originated from protists and bacteria [11], the EST libraries constructed in this study contained much fewer ESTs derived from microorganisms.

### Orthologous genes among three termite species

**Figure 2:**
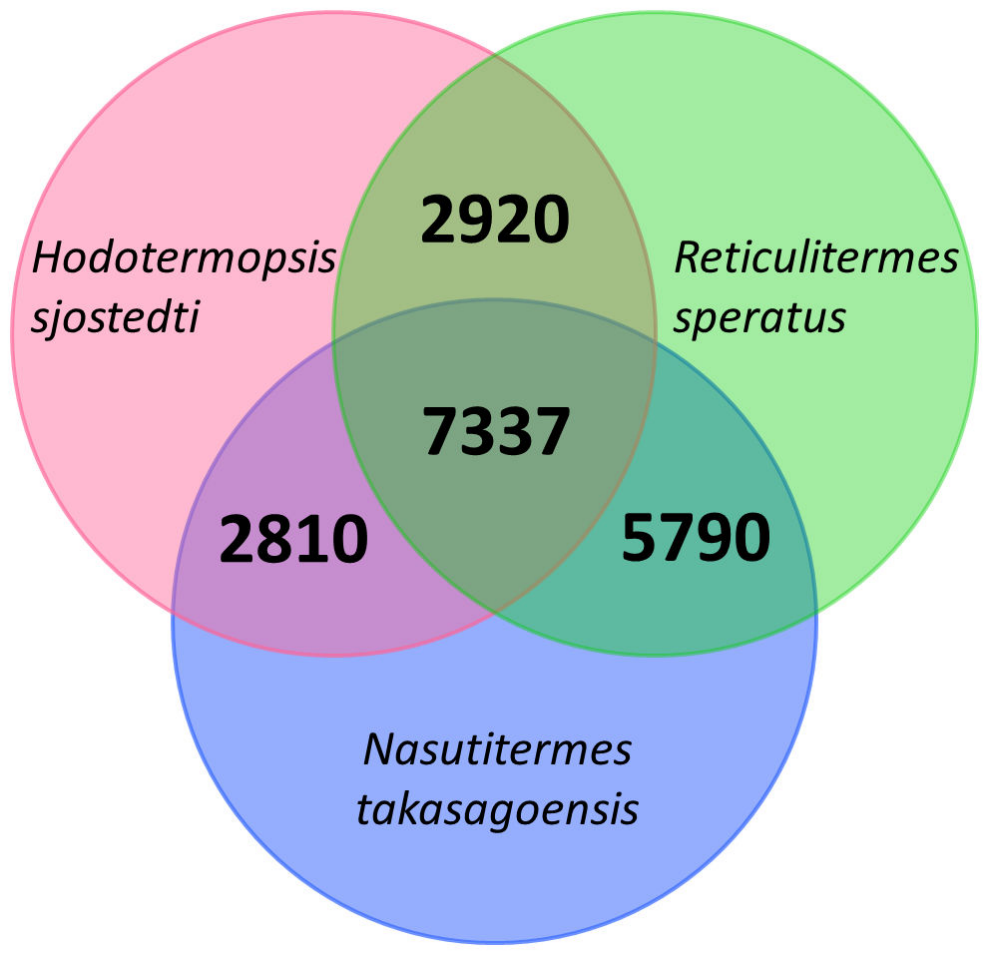
Gene orthology among three termite species. Venn diagram showing overlap of orthologous gene groups among *Hodotermopsis* *sjostedti* , *Reticulitermessperatus* and *Nasutitermestakasagoensis* .

Protein coding regions were predicted and extracted from the isotigs and singletons by OrfPredictor [41], and orthology of the predicted protein sequences among the three termite species was determined by InParanoid [42] and Multiparanoid [43]. As a result, we found 7337 orthologous gene groups that were shared among the three termite species (Figure 2). Among these orthologous gene groups, 377 did not show sequence similarity in the aforementioned BLASTX searches against the nr database. These gene groups without similarity to known genes potentially include novel genes that have evolved in the termite lineage and are widespread among extant termite species. However, to determine whether these genes are unambiguously termite-specific novel genes, it is necessary to examine transcriptome or genome of close relatives of termites such as the wood roach *Cryptocercus* [44], neither of which have been generated so far.

We further examined the putative termite-specific genes by using the results of the InterProScan searches with Pfam database. We found that of the 377 genes seven contained Pfam motifs: two of them had ‘PBP/GOBP family’ (Pfam ID: PF01395) domain and the rest contained ‘EB module’ (PF01683), ‘Regulator of G protein signalling domain’ (PF00615), ‘THAP domain’ (PF05485), ‘Cystatin domain’ (PF00031), and ‘Zinc-finger of C2H2 type’ (PF12874). It is interesting that genes categorized in the PBP/GOBP (Pheromone/general odorant binding protein) family were candidates of the termite-specific novel genes, because, in social insects, pheromonal communication is a very important component of colony organization [45-47]. These PBP/GOBP family proteins might have evolved in association with the new communication functions for social life.

### DNA methylation

We explored the possibility of DNA methylation in the three termite species by examining the presence of DNA methyltransferases (*dnmt1, dnmt*2 and *dnmt*3) and methyl-CpG binding domain (mbd) in the EST libraries. DNMT1 is required to maintain pre-existing methylation patterns in the newly synthesized strand during DNA replication [48]. Although DNMT2 was considered to be a DNA methyltransferase, it is now recognized as a tRNA methyltransferase [49,50]. DNMT3, known as a *de novo* methyltransferase, establishes methylation patterns on unmethylated DNA [51]. MBD proteins have motifs that specifically recognize and are responsible for binding to methyl-CpG [52]. We carried out TBLASTN searches against the EST libraries by using protein sequences of DNMTs and MBDs derived from *Acyrthosiphon pisum*, *Apis mellifera*, *Nasonia* *vitripennis* , *Pediculus* *humanus* and *Tribolium* *castaneum* as query sequences (E-value threshold of 1e-20). The result suggests that all *dnmt*s and *mbd* were present in *H* *. sjostedti* (Table 3). In *R* *. speratus* and *N* *. takasagoensis* , *dnmt1*, *dnmt2*, and *mbd* genes were suggested to be present, while *dnmt3* sequences were not detected at the threshold. However, DNMT3 sequences of *A. mellifera* and *N* *. vitripennis* showed similarity with E values of 2e-19 and 1e-7 to the ESTs of *R* *. speratus* and *N* *. takasagoensis* , respectively. In these two species, a further study is needed to clarify the presence of *dnmt3*. These results suggest occurrence of DNA methylation at least in *H* *. sjostedti* , as suggested in previous studies in rhinotermitid termites, *C* *. lacteus* [53], *C* *. formosanus* and *R* *. flavipes* [54].

**Figure 3:**
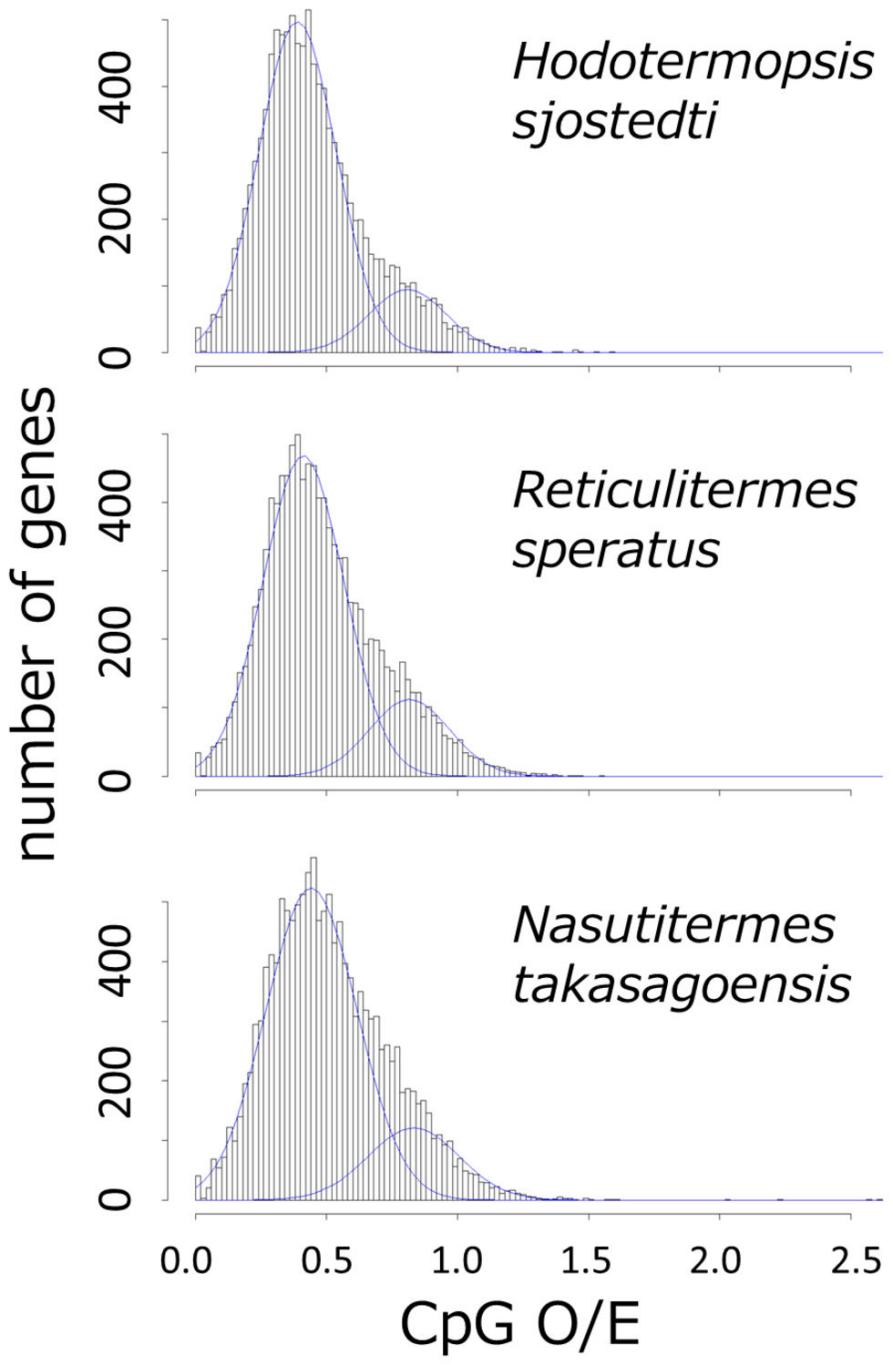
Distribution of normalized CpG content (CpG O/E) in three termite species. Predicted normal curves were fitted to the distributions.

To estimate levels of DNA methylation in coding regions, we calculated CpG O/E of the coding sequences predicted from the isotigs and singletons. We also examined distribution of CpG O/E for bimodality by using NOCOM software [55]. In all of the termite species, the distributions were bimodal, and regarded as mixtures of two normal distributions (log-likelihood ratio test for mono- vs bi-modal distribution model, χ^2^ = 1333.4, *d.f.* = 2, *p* < 0.001 in *H* *. sjostedti* ; χ^2^ = 1056.4, *d.f.* = 2, *p* < 0.001 in *R* *. speratus* ; χ^2^ = 721.6, *d.f.* = 2, *p* < 0.001 in *N* *. takasagoensis* ; Figure 3). The estimated mean ± SD of the two mixed normal distributions were 0.39 ± 0.14 and 0.81 ± 0.14, 0.41 ± 0.15 and 0.82 ± 0.15, and 0.44 ± 0.17 and 0.83 ± 0.17, with the proportion of the former normal distribution of 0.84, 0.81, and 0.81 in *H* *. sjostedti* , *R* *. speratus* and *N* *. takasagoensis* respectively. This result indicates that the majority of the genes showed low CpG O/E values (less than 1). Depletion of CpG O/E and bimodality of its distribution are representative features of methylated genomes [54]. Because methylated cytosines have a tendency to mutate to thymines through deamination, CpG O/E of heavily methylated regions decreases, and instead, TpG O/E and CpA O/E increase over evolutionary time [30]. In the three termite species, the distributions of TpG O/E and CpA O/E were shifted to the right compared to the distributions of the other dinucleotide combinations (Figures S2-S4). This result also supports DNA methylation in the majority of the genes of the three termites.

To characterize coding sequences with relatively low- and high-CpG O/E, we first classified them into either low- or high-CpG O/E class by using the intersection of the two normal curves of CpG O/E obtained by NOCOM analysis as threshold of the classification. Then, we determined enriched GO terms in low- and high-CpG O/E genes. In all of the three species, ‘cellular process’ (GO:0009987), ‘localization’ (GO:0051179) and ‘establishment of localization’ (GO:0051234), all of which are belonging to the BP category, were significantly enriched in the low-CpG O/E class (Figure S5). On the other hand, ‘extracellular region’ (GO:0005576) of the CC category, ‘structural molecule activity’ (GO:0005198), ‘transporter activity’ (GO:0005215), ‘electron carrier activity’ (GO:0009055) and ‘molecular transducer activity’ (GO:0060089) of the MF category were significantly enriched in the high-CpG O/E class. A previous study of DNA methylation [54] showed that ‘extracellular region’ and ‘structural molecule activity’ were enriched in the high-CpG O/E class also in *R* *. flavipes* and in *C* *. formosanus* , respectively. On the other hand, in the low-CpG O/E class, there were no consistent GO categories with the previous study. As suggested in the previous study [54], the inconsistency in GO categories enriched in the high- and low-CpG O/G genes might be due to differences in transcriptome completeness between EST libraries, rather than due to species-specific methylation change over evolutionary time.

## Conclusions

We generated normalized cDNA libraries using almost all castes, sexes and developmental stages, and sequenced them using the 454 GS FLX Titanium system in three species of termites, *Hodotermopsis* *sjostedti* , *Reticulitermessperatus* and *Nasutitermestakasagoensis* . The EST libraries of the three species contained most of the genes conserved among a wide range of taxa, that is, CEGs and genes conserved among the insects registered in OrthoDB6. Genes that are not conserved among the taxa, that is, lineage-specific genes, are also likely to be present in the EST libraries. The result of BLASTX searches with the nr database showed that more than a half of ESTs did not exhibit similarity to known genes. Such ESTs without BLASTX hits may contain lineage-specific genes. Therefore, the EST libraries of the three species of termites are expected to cover most of their transcriptomes and be useful for future molecular biological studies on termites.

Our computational analyses suggested that DNA methylation occurs in the three species of termites. However, it is still unclear whether caste differentiation is influenced by DNA methylation in termites. DNA methylation is a candidate proximate mechanism for generating polyphenism in insects [56,57]; for example, in the honey bee, *Apis mellifera*, DNA methylation was suggested to influence caste development [58]. In two rhinotermitid termites, *C* *. formosanus* and *R* *. flavipes* , CpG O/E was associated with gene expression profile among castes [54]. However, on the other hand, in the termite *C* *. lacteus* , caste-specific DNA methylation was not detected by methylation-sensitive amplified fragment length polymorphism analysis [53]. Thus, further extensive analyses on association between DNA methylation and caste polymorphism are needed to understand social organization from a molecular perspective in termites.

## Methods

### Termite collection

For EST library construction, we used termite individuals derived from 13 colonies of *Hodotermopsis* *sjostedti* that were collected during 2002-2011 from Yakushima Island, Kagoshima Prefecture, Japan. Seven colonies of *Reticulitermessperatus* were collected in 2010 from Toyama and Ishikawa Prefectures, Japan. Three colonies of *Nasutitermestakasagoensis* were collected in 2011 from Iriomote Island, Okinawa Prefecture, Japan. Until RNA extraction or rearing experiments, the termites were maintained with nest materials at room temperature (25±1°C) in the laboratory, except for nymphs of *H* *. sjostedti* , which had been stored at -80°C, and for a king, a queen and N3 nymphs of *N* *. takasagoensis* , which had been stored in RNAlater (Ambion, Austin, TX, USA) at -80 °C (see Tables S1-S3 for detailed description of castes).

No specific permits were required for the described field studies and the locations are not privately-owned or protected in any way. All the three termite species are not endangered or protected species.

### Alate pairing and establishment of incipient colonies

To obtain queens, kings, small presoldiers and small soldiers of *H* *. sjostedti* (see Table S1 for descriptions of these castes), we established incipient colonies by pairing alates, which become kings and queens after dealation and initiation of colonies. Alates derived from two of the collected colonies were paired and reared in plastic boxes (50 mm × 60 mm × 25 mm) filled with decaying wood flakes at room temperature for four months. After four months, kings, queens, small presoldiers and small soldiers were collected and immediately used for RNA extraction.

Similarly, in *R* *. speratus* , to obtain queens, kings, eggs, and larvae, we paired alates derived from two of the field colonies and reared them in glass vials (φ21 mm × 45 mm) filled with decaying wood flakes at room temperature. After seven months from the colony establishment, kings, queens, eggs, and larvae were collected for RNA extraction.

### Induction of presoldier differentiation by juvenile hormone analog treatment

To obtain RNA of pseudergates/workers that are in the process of developing into presoldiers, we carried out presoldier induction experiments with juvenile hormone analogs (JHAs) in *H* *. sjostedti* , *R* *. speratus* , and *N* *. takasagoensis* . For the induction experiments in *H* *. sjostedti* we established experimental colonies, each of which consisted of 10 pseudergates, in a petri dish of 70 mm in diameter with filter paper contained 10 µg of pyriproxyfen; other conditions of the induction were based on Ogino et al. [19]. In *R* *. speratus* , we followed Tsuchiya et al. [21] for presoldier induction and used 80 µg of juvenile hormone III per experimental colony. In *N* *. takasagoensis* , we induced presoldiers with 100 µg of hydroprene according to Toga et al. [59]. Presoldiers induced by JHA were collected for total RNA extraction.

### Induction of neotenic differentiation

To obtain RNA of neotenics, namely nymphoids and ergatoids, of *R* *. speratus* , we induced neotenic differentiation in artificially-established colonies that lacked reproductive individuals. Under some conditions (e.g. in the absence of reproductive individuals), nymphs and workers of *R* *. speratus* differentiate into nymphoids and ergatoids, respectively, through special molt [60,61]. To collect nymphoids, we established artificial colonies, each of which was composed of 20 nymphs, and maintained them as described in Saiki & Maekawa [62]. We collected some nymphoids that appeared in the artificial colonies within 24 hrs from molting, and referred them as ‘newly emerged nymphoid’ (Table S2). The other nymphoids, referred as ‘nymphoid’ (Table S2), were continued to be reared for a while and then collected. To collect ergatoids, we established 10 artificial colonies, each of which was composed of 50 workers and maintained in a plastic box (50 mm × 60 mm × 25 mm) lined with moistened filter paper at 25°C under constant darkness. We collected ergatoids that were differentiated from the workers in the artificial colonies. These nymphoids and ergatoids were used for RNA extraction.

### Total RNA extraction

For RNA extraction, we used termite individuals that were classified into 18, 26, and 25 categories on the basis of their castes/sexes/developmental stages for *H* *. sjostedti* , *R* *. speratus* , and *N* *. takasagoensis* , respectively (Tables S1-S3). Caste and developmental stage were determined based on external morphology and body size, and sex was determined based on morphology of sternites, as described in previous studies (caste and developmental stage: Miura et al. [63,64] for *H* *. sjostedti* , Kawamura [65] and Takematsu [66] for *R* *. speratus* , and Hojo et al. [67] for *N* *. takasagoensis* ; sex determination: Miura et al. [63] for *H* *. sjostedti* , Hayashi et al. [68] for *R* *. speratus* , and Hojo et al. [67] for *N* *. takasagoensis* ).

Before RNA extraction, to reduce contamination of RNA of intestinal symbiotic protozoans, we dissected out and removed the digestive tracts of the termites, except for young-instar larvae, which lack the symbionts, using forceps. For the first purification of total RNA we used combination of ISOGEN and High-Salt Precipitation Solution (Nippon Gene, Toyama, Japan), following the protocol provided by the manufacturer after termites were homogenized in the ISOGEN solution, and obtained total RNA dissolved in 50-100 µl of nuclease-free water. The total RNA was then subjected to DNase treatment and second purification with SV Total RNA Isolation System (Promega, Madison, WI). Finally the total RNA was dissolved in appropriate volumes (80-160 µl) of nuclease-free water.

### Normalized cDNA library construction for 454 pyrosequencing and sequence assembling

Total RNAs from the different castes/sexes/developmental stages of each species were pooled together. The pooled total RNA was then reverse transcribed with M-MLV Reverse Transcriptase (Promega, Madison, WI, USA) and the primer 5’-CAAGCAGAAGACGGCATACGACTGGAG(T) _16_VN-3’ (where V is A, C, or G, and N is any nucleotide) containing a *Gsu*I recognition site. Subsequently, after oxidation of the diole group, biotinylation of the 5’ Cap structure of mRNA, and RNase I treatment, full-length cDNA/RNA hybrids were selectively captured by magnetic beads attaching streptavidin (Streptavidin Sepharose High Performance; GE healthcare, Piscataway, NJ, USA). The RNA was decomposed by incubation in 50 mM NaOH at 37°C and full-length single stranded cDNA was purified. Then, two different, partially double-stranded adaptors (adaptor GN5 and N6), each of which had a *Gsu*I recognition site, were mixed in a ratio of 4:1 and attached to the single strand cDNA by using DNA Ligation Kit, Mighty Mix (Takara, Ohtsu, Japan). The adaptor GN5 was composed of 5'-AATGATACGGCGCTGGAGGACAGGTTCAGAGTTCG(N)_5_-3' and its combining complement 3'-TTACTATGCCGCGACCTCCTGTCCAAGTCTCAAG-5. The adaptor N6 was composed of 5'-AATGATACGGCGCTGGAGGACAGGTTCAGAGTTC(N)_6_-3' and its combining complement 3'-TTACTATGCCGCGACCTCCTGTCCAAGTCTCAAG-5'. Following the adaptor ligation, the second strand cDNA was synthesized with Takara Bio, LA Taq (Takara) and the primer 5'-AATGATACGGCGCTGGAGGACAGGTTCAGAGTTC-3'. To minimize difference in abundance among different transcripts, the double stranded cDNA was normalized. For normalization, the cDNA was first denatured at 98°C for 2 min, hybridized at 68°C for 5 hrs, and digested by a double-strand specific DNA nuclease (Duplex-specific Nuclease, Crab, recombinant, Solution; Wako Pure Chemical Industries, Ltd, Osaka, Japan). The normalized cDNA was subsequently amplified by PCR with the following thermal condition: preheat at 98°C at 1 min, 10 cycles of consecutive 98°C for 10 sec, 55°C at 5 sec and 72°C for 5 min, and final extension at 72°C for 5 min. Finally the PCR products were digested with *Gsu*I for removing the adaptors and poly (A) sequences.

The normalized cDNA library of each termite species was sequenced in a full plate of a Roche 454 FLX Titanium instrument. Sequence assembling was performed using a GS de novo Assembler (Newbler) v2.5.3 (Roche Applied Science, Indianapolis, IN, USA) with cDNA mode (see 454 Sequencing System Software Manual, v2.5.3, Part C, for a detailed description). Newbler explicitly accounts for splice variants in its cDNA mode operation and constructs isotigs. Briefly, Newbler can identify branching structures in multiple alignments of overlapping reads and divides the alignments into multiple contigs within which have no branching conflicts. Then the contigs are grouped into the same isogroups and used for constructing alternative connections among them with a network or graph-based approach. Any contigs or isotigs that share any read overlaps are grouped into the same isogroup. Broadly contigs can be considered as exons, although they may contain untranslated regions (UTRs) and introns, isotigs as splice variants, and isogroups as groups of splice variants that are generated from single genes. All of the isotigs and singletons obtained by the assembling, except for short singletons (<100 bp), were used for the further analyses.

The preparation of normalized cDNA libraries, 454 pyrosequencing and sequence assembling were performed as a custom service by Genaris, Inc (Yokohama, Kanagawa, Japan).

### Functional annotation with non-redundant database, Gene Ontology, and Pfam database

To annotate isotigs and singletons, we carried out sequence similarity searches using BLASTX algorithm (version 2.2.26) [69] against the non-redundant (nr) database (downloaded on 30 Oct 2012) of GenBank. We set E-value threshold of ≤1e-4 and bit score threshold of ≥40 for BLAST hit. Based on the BLASTX top hit genes, Gene Ontology (GO) term IDs were assigned to the isotigs and singletons by using ID mapping data of UniProt (28 Nov 2012 released version, downloaded from ftp://ftp.uniprot.org/pub/databases/uniprot/current_release/knowledgebase/idmapping/idmapping_selected.tab.gz) and a histogram of GO terms was generated with WEGO [70].

Protein motifs and domains were identified for all of the predicted gene models (see “Coding sequence prediction” of “Methods”) by running InterProScan (version 5) [34] with default parameters using known domains from Pfam (Release 26.0).

### Species distribution of BLASTX top hit genes

Based on the results of BLASTX with the nr database, we surveyed species distributions of the top hit genes. For this analysis, we used only one isotig selected randomly from a single isogroup when there were multiple isotigs in a single isogroup, because isotigs from the same isogroups have similar sequences and almost always had the same top-hit genes in BLASTX searches, and thus are redundant for the analysis of species distribution.

### Coding sequence prediction

The coding sequences and their corresponding amino acid sequences were extracted from the isotigs and singletons by using OrfPredictor ( [41]; http://proteomics.ysu.edu/tools/OrfPredictor.html).

### Orthologous gene determination

InParanoid [42] was used for identification of orthologous gene pairs of *H* *. sjostedti* and *N* *. takasagoensis* , *H* *. sjostedti* and *R* *. speratus* , and *N* *. takasagoensis* and *R* *. speratus* . Then MultiParanoid [43] was used to merge them into multiple species orthologous groups. InParanoid and MultiParanoid were executed with default parameters.

### Normalized CpG content (CpG O/E)

For calculation of normalized CpG content (CpG O/E), we used the coding sequences obtained by OrfPredictor. Because our EST libraries contained genes derived from organisms other than the termites, such as intestinal protists, we used only the coding sequences originated from the isotigs and singletons whose top hits of the BLASTX search with the nr database were genes derived from insect species. In addition, when there were multiple coding sequences originating from the same isogroups, we randomly selected and used only one coding sequence from an isogroup to avoid redundancy of the sequences. Finally, short coding sequences (<300 bp) were discarded for this analysis.

The normalized CpG content is defined and calculated by the following formula:

CpG O/E = P_CpG_/P_C_P_G_

where P_CpG_, P_C_, and P_G_ are the frequencies of CpG dinucleotides, C nucleotides, and G nucleotides, respectively. We statistically tested whether the frequency distributions of CpG O/E were bimodal rather than unimodal. For the test of bimodality we estimated means, standard deviations and mixture proportions of two normal distributions, and calculated log-likelihoods of frequency distributions under models of uni- and bi-modal distributions with the NOCOM software (Ott 1979). Chi-square tests were then performed for the log-likelihoods with the statistics G^2^ = 2[ln(*L*_*1*_) -ln(*L*_*2*_)] which approximately follows a chi-square distribution with 2 degrees of freedom.

The coding sequences were classified into low- and high-CpG O/E genes by using the intersection of the two normal curves in the CpG O/E frequency distributions as a threshold of the classification. We then made histograms of GO terms for the two classes of the coding sequences. To find enriched GO terms in the two classes, we performed Fisher’s exact tests for the rate of annotated genes to the rest of all genes in each GO term in the two classes. False discovery rate was controlled at q <0.05 by the Benjamini & Hochberg method [71] for each of BP, CC and MF categories.

### Database search for *dnmt* and *mbd* gene sequences

TBLASTN searches were performed for examining presence of DNA (cytosine-5) methyltransferase genes, *dnmt1*, *dnmt2* and *dnmt3*, and methyl-CpG-binding domain, *mbd*, in the EST libraries. We used DNMT and MBD protein sequences of the following insect species downloaded from GenBank as query sequences for the TBLASTN searches against the EST libraries: *Acyrthosiphon pisum* (DNMT1, XP_003243626; DNMT2, XP_001949338; DNMT3a, XP_003241627; DNMT3b, XP_003240668; MBD, NP_001156167), *Apis mellifera* (DNMT1a, NP_001164522; DNMT1b, XP_001122269; DNMT2, XP_393991; DNMT3, NP_001177350; MBD isoform 1, XP_003250633; MBD isoform 2, XP_003250634; MBD isoform 3, XP_392422), *Nasonia* *vitripennis* (DNMT1a, NP_001164521; DNMT1b, XP_001600175; DNMT1c, XP_001607336; DNMT2, XP_001602026; DNMT3, XP_001599223; MBD isoform A, NP_001164526; MBD isoform B, NP_001164527), *Pediculus* *humanus* (DNMT1a, XP_002432160; DNMT1b, XP_002431878; DNMT2, XP_002432555; MBD, XP_002428735) and *Tribolium* *castaneum* (DNMT1, XP_001814230; DNMT2, EFA09160; MBD, XP_969537). E value of ≤1e-20 was set as a threshold for significant hit of the TBLASTN searches.

## Supporting Information

Figure S1 **Caste developmental pathways.** (a) *Hodotermopsis* *sjostedti* , (b) *Reticulitermessperatus* , and (c) *Nasutitermestakasagoensis* . Each arrow indicates a molt. Dotted lines indicate potential molts, which are suggested to occur under natural conditions. It is known that ergatoids are differentiated from workers in *R* *. speratus* , and from workers or larvae in *N* *. takasagoensis* , while instars that have the potential to develop into ergatoids have not been identified.
(PDF)

Figure S2 **Histograms of normalized contents of dinucleotides in *Hodotermopsis* *sjostedti* **.
(PDF)

Figure S3 **Histograms of normalized contents of dinucleotides in *Reticulitermessperatus* **.
(PDF)

Figure S4 **Histograms of normalized contents of dinucleotides in *Nasutitermestakasagoensis* **.
(PDF)

Figure S5 **Frequency and percentage of high- and low-CpG genes annotated by Gene Ontology terms in three termite species.** The terms in which significant differences in frequencies between high- and low-CpG genes were found are indicated by asterisks.
(PDF)

Table S1 **Summary of samples used for cDNA library construction in *Hodotermopsis* *sjostedti* .** Caste, sex, and description of samples, number of individuals, and field colonies from which termite samples originated are shown.
(PDF)

Table S2 **Summary of samples used for cDNA library construction in *Reticulitermessperatus* .** Caste, sex, and description of samples, number of individuals, and field colonies from which termite samples originated are shown.
(PDF)

Table S3 **Summary of samples used for cDNA library construction in *Nasutitermestakasagoensis* .** Caste, sex, and description of samples, number of individuals, and field colonies from which termite samples originated are shown.
(PDF)

Table S4 **Summary of protein domain search result in EST libraries of three termite species.** The 30 most frequently occurring Pfam domains/families in the isotigs and singletons of the three termite species are shown.
(PDF)

Table S5 **Distribution of BLASTX top hits in EST libraries of three termite species.**
(PDF)
