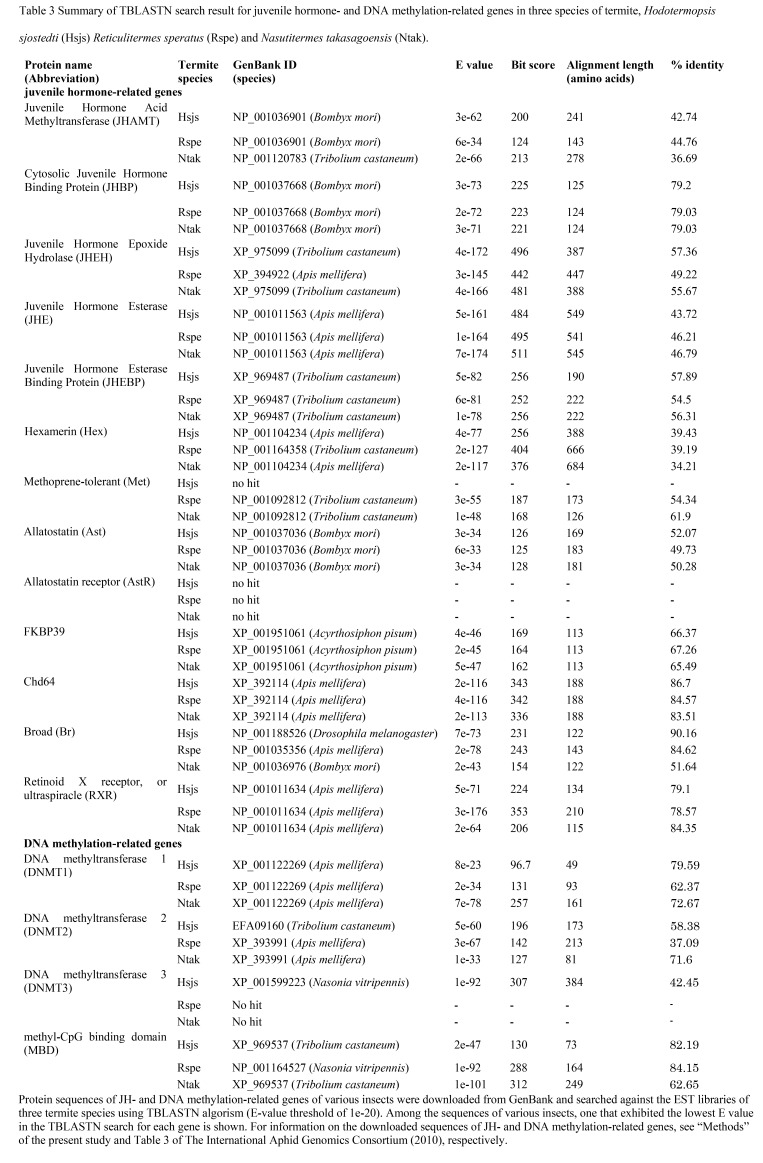# Correction: Construction and Characterization of Normalized cDNA Libraries by 454 Pyrosequencing and Estimation of DNA Methylation Levels in Three Distantly Related Termite Species

**DOI:** 10.1371/annotation/b1ec420f-0227-4362-bb96-638a352f86d4

**Published:** 2013-10-21

**Authors:** Yoshinobu Hayashi, Shuji Shigenobu, Dai Watanabe, Kouhei Toga, Ryota Saiki, Keisuke Shimada, Thomas Bourguignon, Nathan Lo, Masaru Hojo, Kiyoto Maekawa, Toru Miura

Table 3 was incorrectly omitted from the article. It is available here: 

**Figure:**