# Supplementary figures and images for: Construction and Characterization of Normalized cDNA Libraries by 454 Pyrosequencing and Estimation of DNA Methylation Levels in Three Distantly Related Termite Species
Source: PLoS One. 2013 Sep 30;8(9):e76678. doi: 10.1371/journal.pone.0076678 (PMC3787108; doi:10.1371/journal.pone.0076678)

(a)

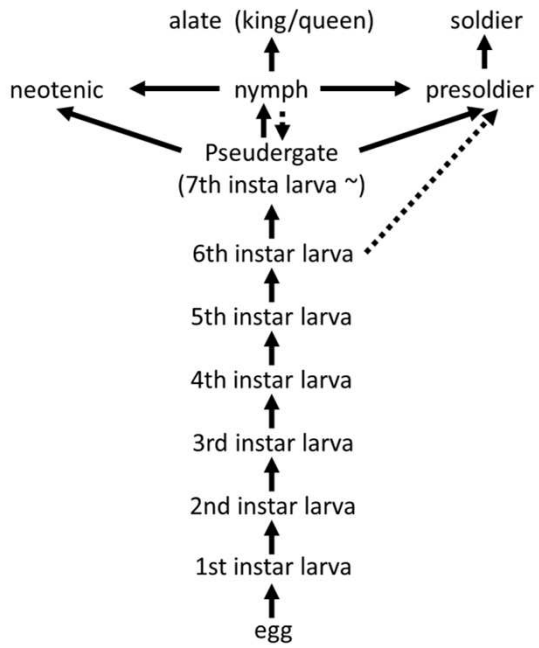

(b)

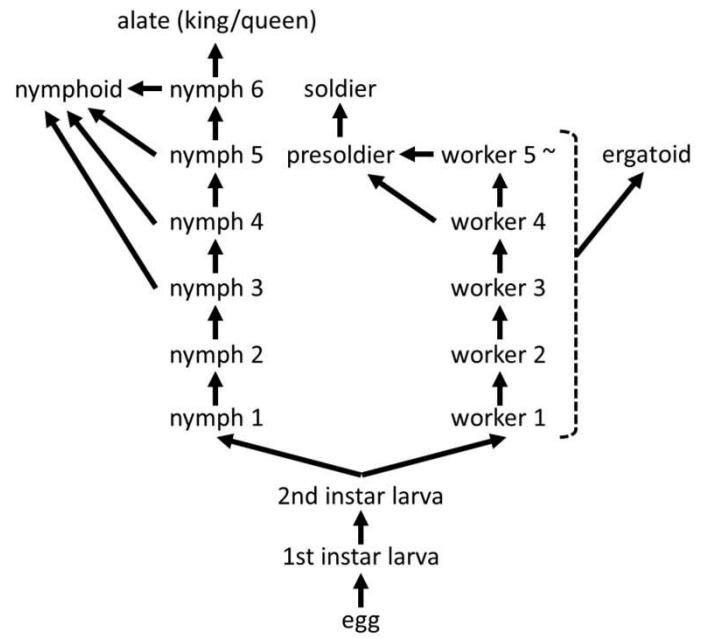

(c)

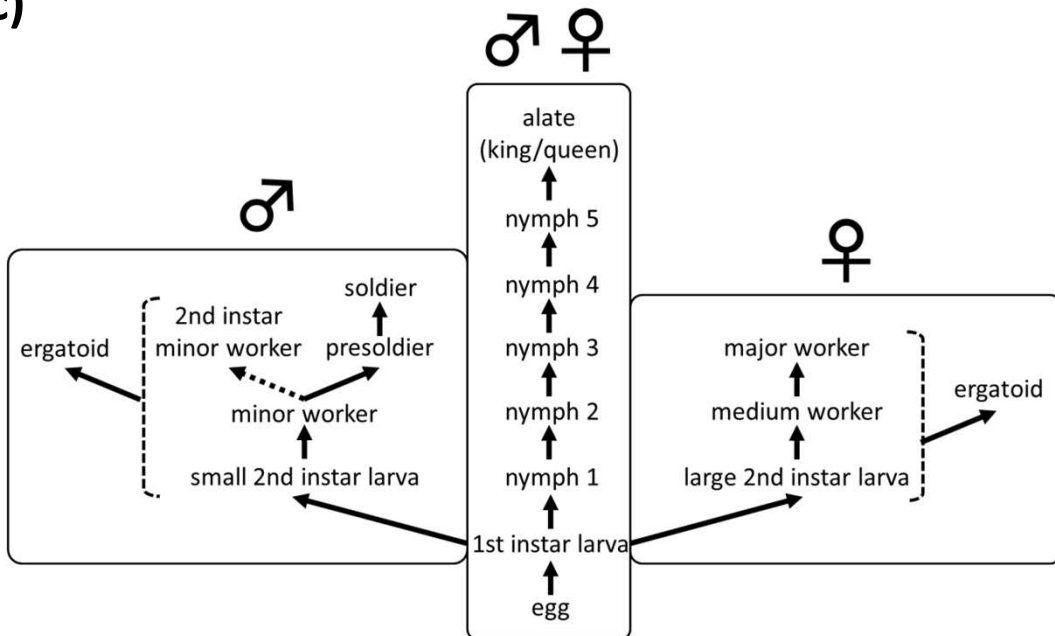

Supplement: Figure S1 — Caste developmental pathways. (a) Hodotermopsis sjostedti , (b) Reticulitermessperatus , and (c) Nasutitermestakasagoensis . Each arrow indicates a molt. Dotted lines indicate potential molts, which are suggested to occur under natural conditions. It is known that ergatoids are differentiated from workers in R . speratus , and from workers or larvae in N . takasagoensis , while instars that have the potential to develop into ergatoids have not been identified. (PDF) [file pone.0076678.s001.pdf]

number of genes

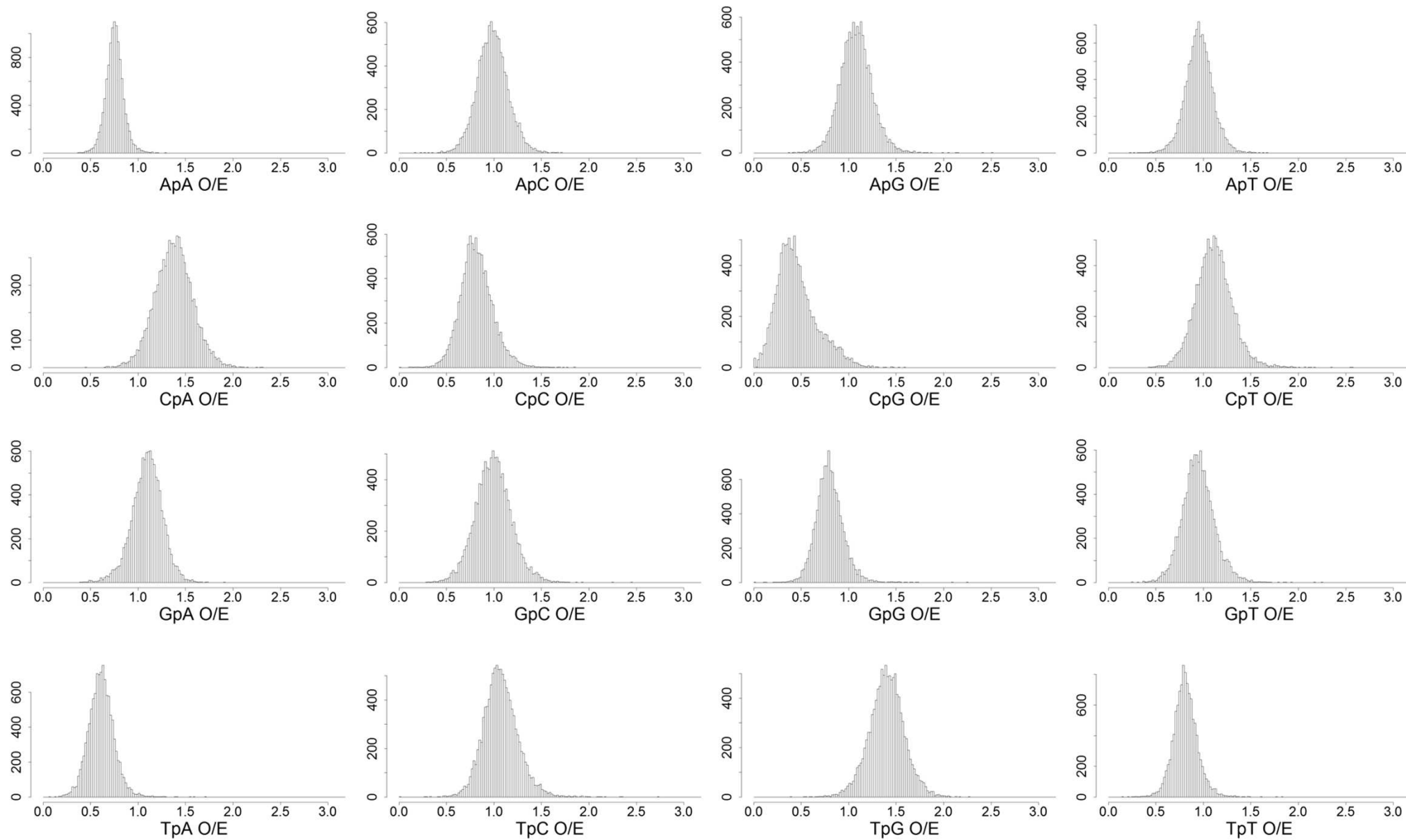

Supplement: Figure S2 — Histograms of normalized contents of dinucleotides in Hodotermopsis sjostedti . (PDF) [file pone.0076678.s002.pdf]

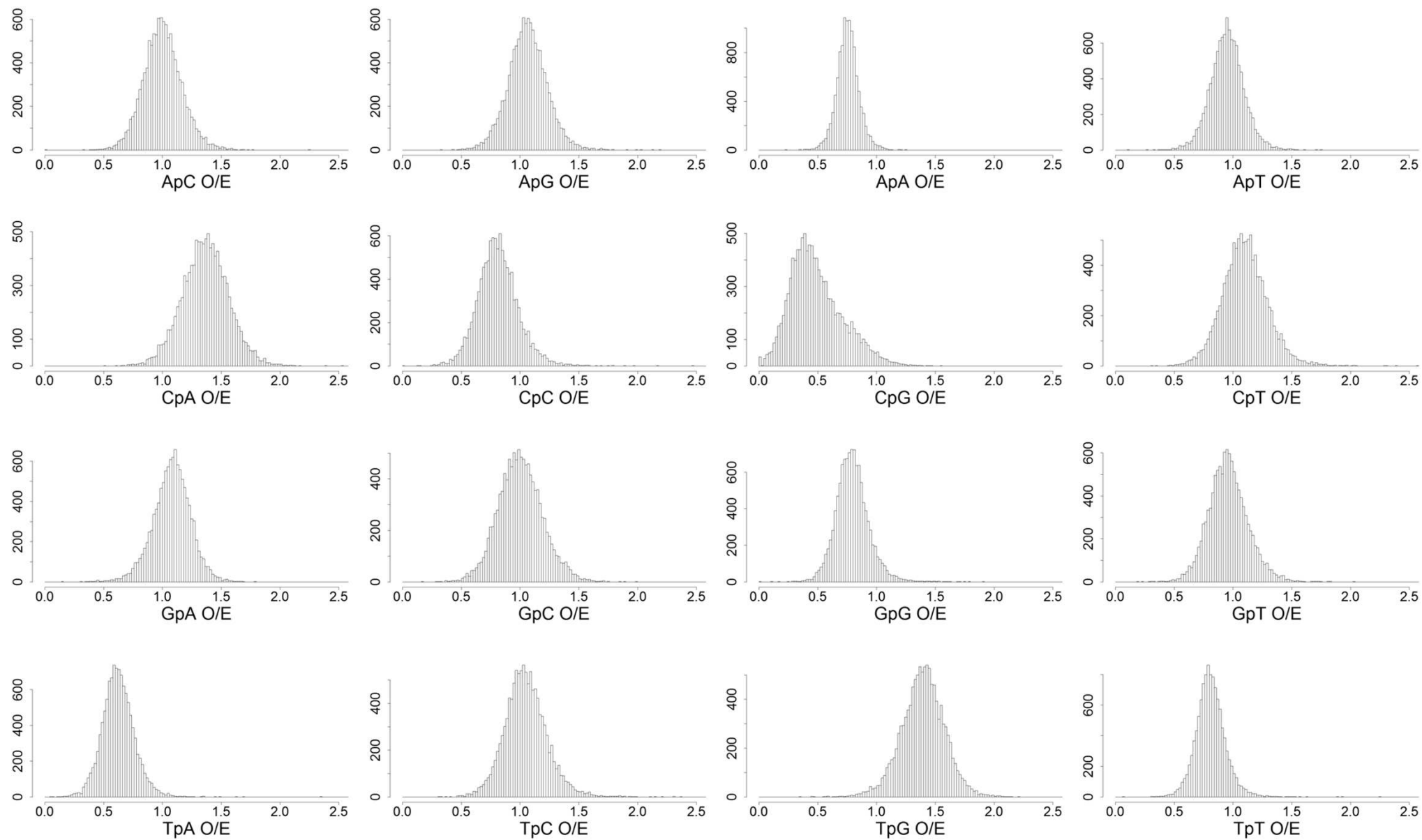

Supplement: Figure S3 — Histograms of normalized contents of dinucleotides in Reticulitermessperatus . (PDF) [file pone.0076678.s003.pdf]

number of genes

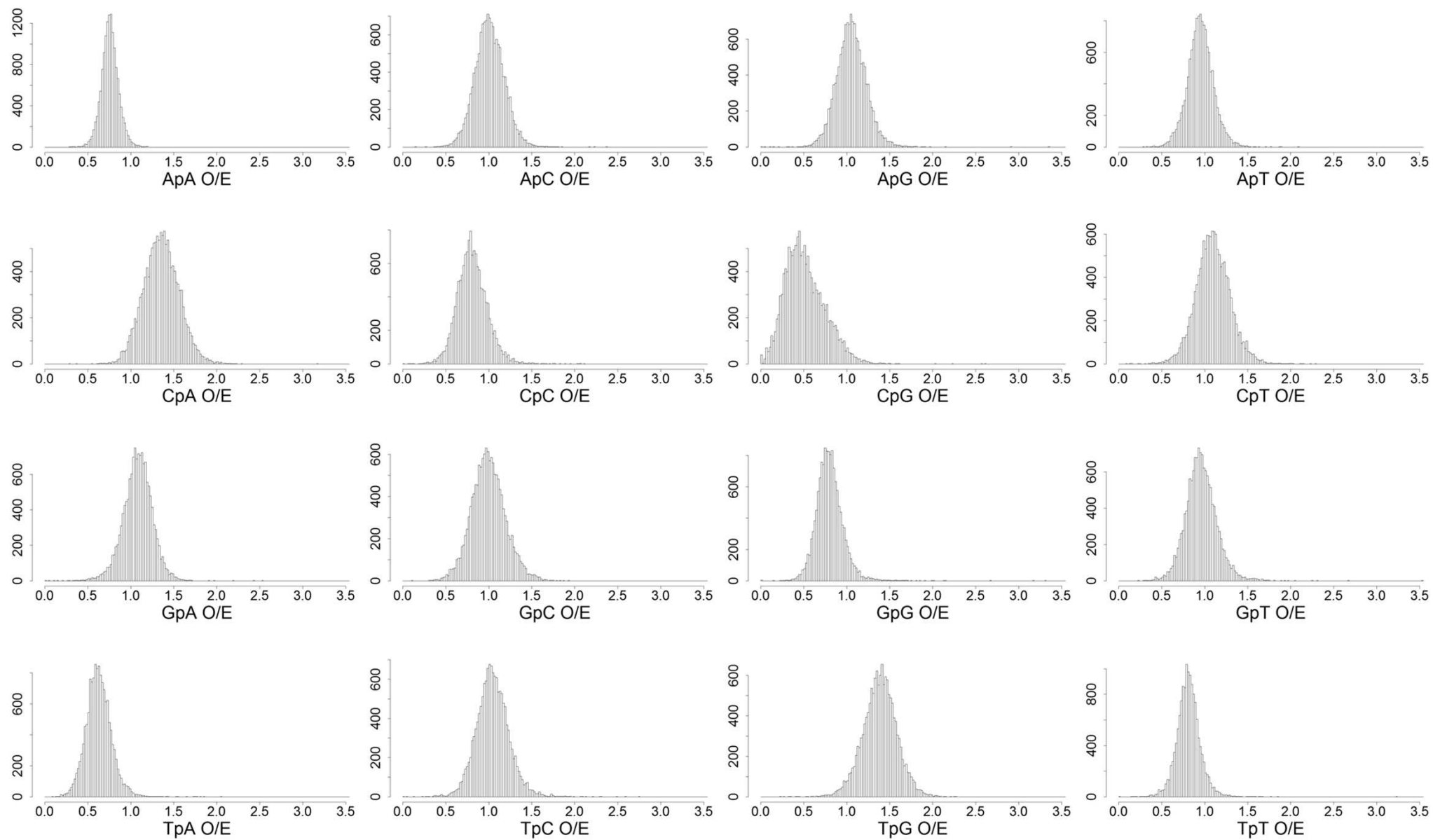

Supplement: Figure S4 — Histograms of normalized contents of dinucleotides in Nasutitermestakasagoensis . (PDF) [file pone.0076678.s004.pdf]

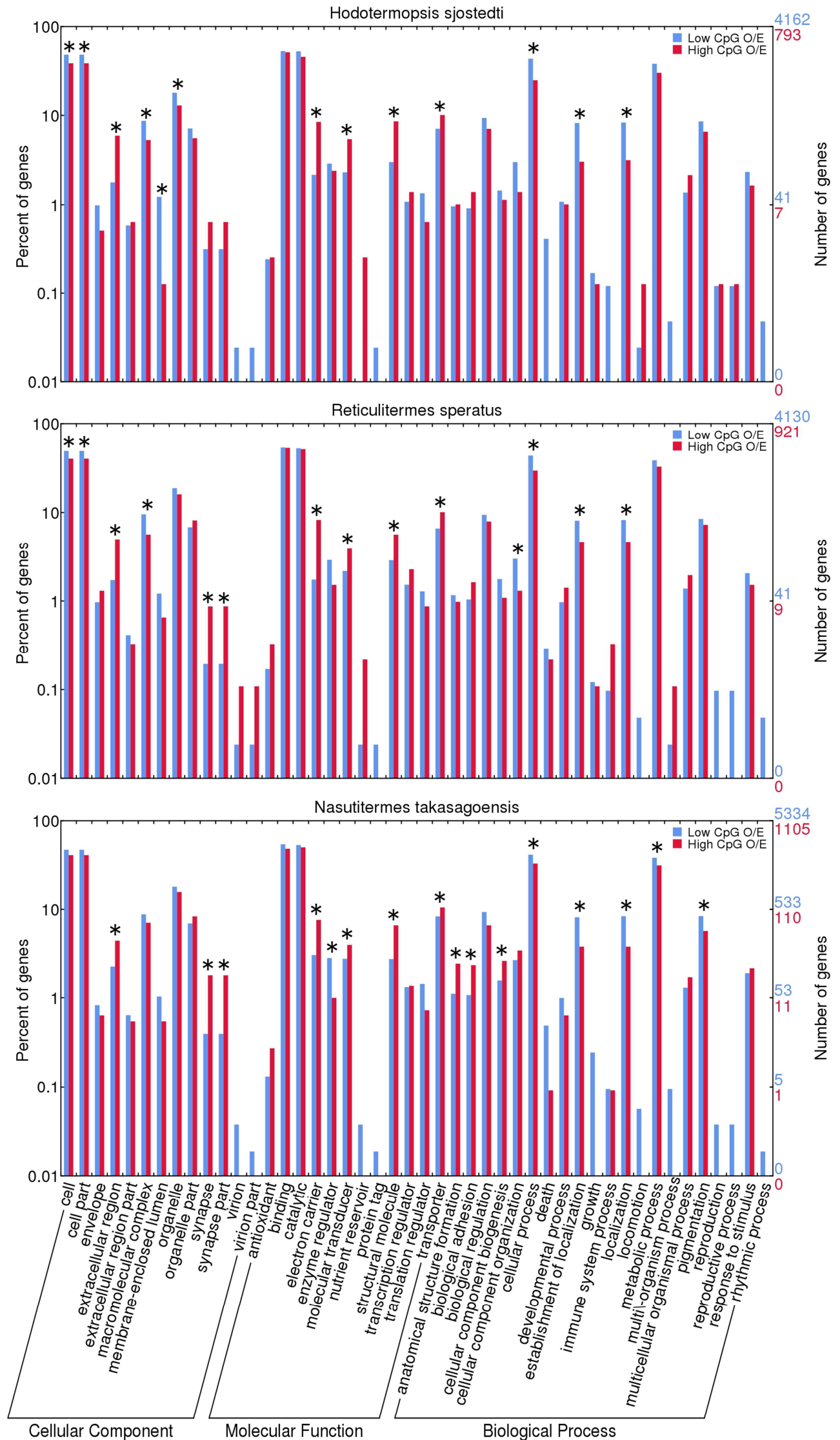

Supplement: Figure S5 — Frequency and percentage of high- and low-CpG genes annotated by Gene Ontology terms in three termite species. The terms in which significant differences in frequencies between high- and low-CpG genes were found are indicated by asterisks. (PDF) [file pone.0076678.s005.pdf]
