# Supplementary material for: Construction and Characterization of Normalized cDNA Libraries by 454 Pyrosequencing and Estimation of DNA Methylation Levels in Three Distantly Related Termite Species
Source: PLoS One. 2013 Sep 30;8(9):e76678. doi: 10.1371/journal.pone.0076678 (PMC3787108; doi:10.1371/journal.pone.0076678)
Supplement: Table S1 — Summary of samples used for cDNA library construction in Hodotermopsis sjostedti . Caste, sex, and description of samples, number of individuals, and field colonies from which termite samples originated are shown. (PDF) [file pone.0076678.s006.pdf]

Table S1. Summary of samples used for cDNA library construction in *Hodotermopsis sjostedti*.

Caste, sex, and description of samples, number of individuals, and field colonies from which termite samples originated are shown.

category ID	caste	sex	description	n	Colony
1	alate	male	winged adults before swarming	4	I, J
2	alate	female	winged adults before swarming	3	J
3	egg	unidentified	embryos of various developmental stages	178 mg[†]	A
4	larva	unidentified	1st-6th instar individuals	57	H, I, K
5	nymph	male and female	immature individuals with wing buds	20	M
6	pseudergate	male and female	7th and greater immature individuals without wings and wing buds and any soldier-like characters	11	E, F, G, H, K
7	soldier	male and female	individuals with sclerotized heads and elongated mandibles for defense	9	F, G, H, J, K, L
8	presoldier	male and female	individuals developmentally anteceding soldiers	2	A, B
9	neoteinc	male	reproductives possessing some juvenile characters	6	D, F, I
10	neotenic	female	reproductives possessing some juvenile characters	7	C, F, H, I
11	king (primary reproductive)	male	kings collected from 4 month-old colonies that were founded artificially by pairing alates[*]	6	I, J
12	queen (primary reproductive)	female	queens collected from 4 month-old colonies that were founded artificially by pairing alates[*]	6	J
13	small soldiers	male and female	small soldiers collected from 4 month-old colonies that were founded artificially by pairing alates[*]	8	I, J
14	small presoldiers	male and female	small presoldiers collected from 4 month-old colonies that were founded artificially by pairing alates[*]	2	I, J

15	JHA-treated pseudergate	male and female	pseudergates that experienced pyriproxyfen application for 7 days*	10	G, H, I, L
16	JHA-treated pseudergate	male and female	pseudergates that experienced pyriproxyfen application for 7 days*	20	G, H, I, J, L
17	JHA-induced presoldier	male and female	presoldiers that were differentiated from pseudergates by pyriproxyfen application in 3-week old artificially established colonies*	14	G, H, I, L
18	JHA-induced presoldier	male and female	presoldiers that were differentiated from pseudergates by pyriproxyfen application in 4-week old artificially established colonies*	8	G, H, I, L

The terms to designate the castes followed Miura et al. (2004).

*For more detailed method of artificial colony establishment and sampling, see “Materials and Methods”.

†All eggs were weighed together.

Reference

Miura T, Koshikawa S, Machida M, Matsumoto T (2004) Comparative studies on alate wing formation in two related species of rotten-wood termites: *Hodotermopsis sjostedti* and *Zootermopsis nevadensis* (Isoptera: Termopsidae). Insectes Soc 51: 247-252.
