# Supplementary material for: Construction and Characterization of Normalized cDNA Libraries by 454 Pyrosequencing and Estimation of DNA Methylation Levels in Three Distantly Related Termite Species
Source: PLoS One. 2013 Sep 30;8(9):e76678. doi: 10.1371/journal.pone.0076678 (PMC3787108; doi:10.1371/journal.pone.0076678)
Supplement: Table S2 — Summary of samples used for cDNA library construction in Reticulitermessperatus . Caste, sex, and description of samples, number of individuals, and field colonies from which termite samples originated are shown. (PDF) [file pone.0076678.s007.pdf]

Table S2. Summary of samples used for cDNA library construction in *Reticulitermes speratus*.

Caste, sex, and description of samples, number of individuals, and field colonies from which termite samples originated are shown.

category ID	caste	sex	description	n	colony
1	egg	unidentified	embryos of various developmental stages, collected from 7 month-old colonies that were founded artificially by pairing alates*	263	A
2	larva	unidentified	1st and 2nd instar individuals, collected from 7 month-old colonies that were founded artificially by pairing alates* and a natural colony	100	A
3	nymph 1	male	3rd instars with wing buds	20	C, D
4	nymph 1	female	3rd instars with wing buds	33	C, D
5	nymph 2	male	4th instars with wing buds	22	C, D
6	nymph 2	female	4th instars with wing buds	24	C, D
7	nymph 3	male	5th instars with wing buds	25	C, D, E
8	nymph 3	female	5th instars with wing buds	25	C, D, E
9	nymph 4	male	6th instars with wing buds	25	E
10	nymph 4	female	6th instars with wing buds	25	E
11	nymph 5	male	7th instars with wing buds	25	E
12	nymph 5	female	7th instars with wing buds	16	E
13	nymph 6	male	8th instars with wing buds	25	E
14	nymph 6	female	8th instars with wing buds	25	E
15	worker 1-4	male and female	3rd-6th instars without wing buds	180	C, D
16	worker 5-	male and female	7th and greater instars without wing buds	50	C
17	soldier	male and female	individuals with sclerotized heads and elongated mandibles for defense	50	E, F, H
18	JH-induced Presoldier	male and female	presoldiers that existed in 2-week colonies with juvenile hormone III†	50	C
19	nymphoid	male	nymph-derived neotenic, possessing some juvenile characters, collected from	6	E

			artificially-established colonies*		
20	nymphoid	female	nymph-derived neotenic, possessing some juvenile characters, collected from artificially-established colonies*	37	E
21	newly emerged nymphoid	male	nymphoids within 24 hrs after emergence, collected from artificially-established colonies*	14	E
22	newly emerged nymphoid	female	nymphoids within 24 hrs after emergence, collected from artificially-established colonies*	9	G
23	ergatoid	male	worker-derived neotenic, possessing some juvenile characters, collected from artificially-established colonies*	5	B
24	ergatoid	female	worker-derived neotenic, possessing some juvenile characters, collected from artificially-established colonies*	31	B
25	king (primary reproductive)	male	adults, collected from 4 month-old colonies that were founded artificially by pairing alates*	20	A
26	queen (primary reproductive)	female	adults, collected from 4 month-old colonies that were founded artificially by pairing alates*	20	A

The terms to designate the castes followed Takematsu (1997) and Roisin (2000)

*For more detailed method of artificial colony establishment and sampling, see “Materials and Methods”.

References

Roisin Y (2000) Diversity and evolution of caste patterns. In: *Termites: evolution, sociality, symbioses, ecology.* In: Abe T, Bignell DE, Higashi M, editors. Termites: evolution, sociality, symbioses, ecology. Dordrecht, Netherlands: Kluwer Academic Publishers. pp. 95-119.

Takematsu Y (1992) Biometrical study on the development of the castes in *Reticulitermes speratus* (Isoptera, Rhinotermitidae). Jpn J Entomol 60: 67-76
