# Supplementary material for: Construction and Characterization of Normalized cDNA Libraries by 454 Pyrosequencing and Estimation of DNA Methylation Levels in Three Distantly Related Termite Species
Source: PLoS One. 2013 Sep 30;8(9):e76678. doi: 10.1371/journal.pone.0076678 (PMC3787108; doi:10.1371/journal.pone.0076678)
Supplement: Table S3 — Summary of samples used for cDNA library construction in Nasutitermestakasagoensis . Caste, sex, and description of samples, number of individuals, and field colonies from which termite samples originated are shown. (PDF) [file pone.0076678.s008.pdf]

Table S3. Summary of samples used for cDNA library construction in *Nasutitermes takasagoensis*.

Caste, sex, and description of samples, number of individuals, and field colonies from which termite samples originated are shown.

category ID	caste	sex	description	n	colony
1	egg	unidentified	embryos at various developmental stages	156 mg[‡]	A
2	larva	unidentified	1st instar undifferentiated individuals	100	A
3	2nd instar	unidentified	2nd instar individuals	90	A
4	nymph 3	male	4th instar individuals with wing buds	12	B
5	nymph 3	female	4th instar individuals with wing buds	2	B
6	nymph 4	male	5th instar individuals with wing buds	10	A
7	nymph 4	female	5th instar individuals with wing buds	10	A
8	nymph 5	male	6th instar individuals with wing buds	10	A
9	nymph 5	female	6th instar individuals with wing buds	10	A
10	nymph 5	male	6th instar individuals with wing buds	6	A
11	nymph 5	female	6th instar individuals with wing buds	6	A
12	alate	male	winged adults shortly after the molting from nymphs	12	A
13	alate	female	winged adults shortly after the molting from nymphs	6	A
14	alate	male	winged adults just before swarm	6	A
15	alate	female	winged adults just before swarm	6	A
16	king	male	a mature king collected from a mature colony	1	C
17	queen	female	a mature, physogastric queen collected from a mature colony	1	C
18	worker	male and female	3rd and greater instars of apterous individuals, including minor, medium, and major workers	80	A
19	minor worker	male[*]	3rd-instar individuals without wings and wing buds and any soldier-like characters	100	A
20	JHA-treated minor worker	male[*]	minor workers that experienced hydroprene application for 7 days[†]	240	A
21	JHA-treated	male[*]	minor workers that experienced hydroprene application for 14 days[†]	254	A

	minor worker				
22	JHA-treated minor worker	male*	minor workers that experienced hydroprene application for 21 days†	95	A
23	presoldier	male*	individuals developmentally anteceding soldiers	54	A
24	JHA-induced Presoldier	male*	presoldiers that existed in 2-week colonies with hydroprene†	5	A
25	soldier	male*	individuals with sclerotized heads and nasutes for defense	90	A

The terms to designate the castes followed Hojo et al. (2004) for worker castes and Watson and Abbey (1977) for nymph castes.

*Because sex of the minor workers, presoldiers and soldiers are all male in *Nasutitermes* according to Noirot (1955), sex of them was not checked in this study.

†For more detailed method of artificial colony establishment and sampling, see "Materials and Methods".

‡All eggs were weighed together.

References

Hojo M, Koshikawa S, Matsumoto T, Miura T (2004) Developmental pathways and plasticity of neuter castes in *Nasutitermes takasagoensis* (Isoptera: Termitidae). Sociobiology 44: 433-441.

Noirot C (1955) Recherches sur le polymorphisme des termites superieurs (Termitidae). Ann des Sci Nat Zool 17: 399-595.

Watson. JAL, Abbey HM (1977) The development of reproductives in *Nasutitennes exitiosus* (Hill) (lsoptera: Termitidae). J Aust Ent Soc 16: 161-164.
