# Supplementary material for: Construction and Characterization of Normalized cDNA Libraries by 454 Pyrosequencing and Estimation of DNA Methylation Levels in Three Distantly Related Termite Species
Source: PLoS One. 2013 Sep 30;8(9):e76678. doi: 10.1371/journal.pone.0076678 (PMC3787108; doi:10.1371/journal.pone.0076678)
Supplement: Table S4 — Summary of protein domain search result in EST libraries of three termite species. The 30 most frequently occurring Pfam domains/families in the isotigs and singletons of the three termite species are shown. (PDF) [file pone.0076678.s009.pdf]

Table S4. Species distribution of BLASTX top hits in EST libraries of three termite species.

Hodotermopsis sjostedti			*Reticulitermes speratus*			*Nasutitermes takasagoensis*		
species	# hits	% hits	species	# hits	% hits	species	# hits	% hits
Tribolium castaneum	2788	11.7	*Tribolium castaneum*	2894	11.8	*Tribolium castaneum*	4256	11.8
Pediculus humanus	2562	10.7	*Pediculus humanus*	2645	10.8	*Pediculus humanus*	3935	10.9
Nasonia vitripennis	1276	5.4	*Nasonia vitripennis*	1320	5.4	*Acyrthosiphon pisum*	1974	5.5
Camponotus floridanus	1199	5.0	*Megachile rotundata*	1200	4.9	*Nasonia vitripennis*	1847	5.1
Megachile rotundata	1155	4.8	*Acyrthosiphon pisum*	1143	4.7	*Megachile rotundata*	1752	4.8
Acyrthosiphon pisum	1120	4.7	*Camponotus floridanus*	1117	4.6	*Camponotus floridanus*	1696	4.7
Harpegnathos saltator	884	3.7	*Harpegnathos saltator*	938	3.8	*Harpegnathos saltator*	1654	4.6
Acromyrmex echinatior	701	2.9	*Acromyrmex echinatior*	718	2.9	*Acromyrmex echinatior*	1161	3.2
Trichomonas vaginalis	652	2.7	*Bombus impatiens*	605	2.5	*Bombus impatiens*	920	2.5
Bombus impatiens	623	2.6	*Danaus plexippus*	602	2.5	*Danaus plexippus*	908	2.5
Danaus plexippus	584	2.4	*Apis mellifera*	574	2.3	*Daphnia pulex*	781	2.2
Apis mellifera	518	2.2	*Daphnia pulex*	524	2.1	*Apis mellifera*	773	2.1
Apis florea	514	2.2	*Apis florea*	522	2.1	*Apis florea*	767	2.1
Solenopsis invicta	511	2.1	*Solenopsis invicta*	507	2.1	*Aedes aegypti*	742	2.1
Bombus terrestris	503	2.1	*Bombus terrestris*	465	1.9	*Solenopsis invicta*	738	2.0
Daphnia pulex	477	2.0	*Aedes aegypti*	439	1.8	*Bombus terrestris*	714	2.0
Aedes aegypti	435	1.8	*Culex quinquefasciatus*	321	1.3	*Culex quinquefasciatus*	505	1.4
Anopheles gambiae	323	1.4	*Anopheles gambiae*	307	1.3	*Anopheles gambiae*	466	1.3
Culex quinquefasciatus	308	1.3	*Branchiostoma floridae*	263	1.1	*Bombyx mori*	419	1.2
Branchiostoma floridae	287	1.2	*Trichomonas vaginalis*	251	1.0	*Branchiostoma floridae*	380	1.1
others	6429	27.0	others	7194	29.3	others	9736	27.0
total	23849	100.0	total	24549	100.0	total	36124	100.0
