# Supplementary material for: Construction and Characterization of Normalized cDNA Libraries by 454 Pyrosequencing and Estimation of DNA Methylation Levels in Three Distantly Related Termite Species
Source: PLoS One. 2013 Sep 30;8(9):e76678. doi: 10.1371/journal.pone.0076678 (PMC3787108; doi:10.1371/journal.pone.0076678)
Supplement: Table S5 — Distribution of BLASTX top hits in EST libraries of three termite species. (PDF) [file pone.0076678.s010.pdf]

Table S5. Summary of protein domain search result in EST libraries of three termite species.

The 30 most frequently occurring Pfam domains/families in the isotigs and singletons of the three termite species are shown.

Hodotermopsis sjostedti			*Reticulitermes speratus*			*Nasutitermes takasagoensis*		
Conserved domain/family	Accession ID	No. of hits	Conserved domain/family	Accession ID	No. of hits	Conserved domain/family	Accession ID	No. of hits
Zinc-finger double domain	PF13465	1050	Zinc-finger double domain	PF13465	955	Zinc-finger double domain	PF13465	1303
WD domain, G-beta repeat	PF00400	399	WD domain, G-beta repeat	PF00400	471	WD domain, G-beta repeat	PF00400	440
Zinc finger, C2H2 type	PF00096	260	Zinc finger, C2H2 type	PF00096	234	Zinc finger, C2H2 type	PF00096	318
Cytochrome P450	PF00067	212	Protein kinase domain	PF00069	224	Cytochrome P450	PF00067	254
Protein kinase domain	PF00069	202	Cytochrome P450	PF00067	190	Protein kinase domain	PF00069	224
Ankyrin repeats (3 copies)	PF12796	121	Ankyrin repeats (3 copies)	PF12796	189	Reverse transcriptase (RNA-dependent DNA polymerase)	PF00078	167
RNA recognition motif. (a.k.a. RRM, RBD, or RNP domain)	PF00076	107	RNA recognition motif. (a.k.a. RRM, RBD, or RNP domain)	PF00076	153	Leucine rich repeat	PF13855	143
Ras family	PF00071	96	Leucine rich repeat	PF13855	123	RNA recognition motif. (a.k.a. RRM, RBD, or RNP domain)	PF00076	141
C2H2-type zinc finger	PF13894	92	Reverse transcriptase (RNA-dependent DNA polymerase)	PF00078	119	Ankyrin repeats (3 copies)	PF12796	140
Leucine rich repeat	PF13855	91	Ras family	PF00071	104	Trypsin	PF00089	126
Immunoglobulin I-set domain	PF07679	88	Mitochondrial carrier protein	PF00153	91	Mitochondrial carrier protein	PF00153	94
Mitochondrial carrier protein	PF00153	79	Immunoglobulin I-set	PF07679	76	C2H2-type zinc finger	PF13894	89

			domain					
C2H2-type zinc finger	PF13912	76	Trypsin	PF00089	73	Sugar (and other) transporter	PF00083	89
EF-hand domain pair	PF13499	72	C2H2-type zinc finger	PF13894	71	Immunoglobulin I-set domain	PF07679	85
Zinc-finger associated domain (zf-AD)	PF07776	69	Zinc-finger associated domain (zf-AD)	PF07776	71	Transposase IS4	PF13843	85
BTB/POZ domain	PF00651	68	DEAD/DEAH box helicase	PF00270	65	Ras family	PF00071	80
short chain dehydrogenase	PF00106	67	EF-hand domain pair	PF13499	63	C2H2-type zinc finger	PF13912	79
Carboxylesterase family	PF00135	66	TPR repeat	PF13414	63	Insect cuticle protein	PF00379	73
Reverse transcriptase (RNA-dependent DNA polymerase)	PF00078	63	BTB/POZ domain	PF00651	62	UDP-glucoronosyl and UDP-glucosyl transferase	PF00201	72
Transposase IS4	PF13843	62	Sugar (and other) transporter	PF00083	56	Zinc-finger associated domain (zf-AD)	PF07776	72
DDE superfamily endonuclease	PF03184	57	C2H2-type zinc finger	PF13912	55	BTB/POZ domain	PF00651	71
DEAD/DEAH box helicase	PF00270	57	Carboxylesterase family	PF00135	55	short chain dehydrogenase	PF00106	71
Glycosyl hydrolase family 7	PF00840	57	Haemolymph juvenile hormone binding protein (JHBP)	PF06585	55	Chitin binding Peritrophin-A domain	PF01607	64
Trypsin	PF00089	57	Insect cuticle protein	PF00379	54	TPR repeat	PF13414	61
Chitin binding Peritrophin-A domain	PF01607	56	short chain dehydrogenase	PF00106	54	Carboxylesterase family	PF00135	58
Sugar (and other) transporter	PF00083	55	Leucine Rich Repeat	PF00560	53	EF-hand domain pair	PF13499	57
AMP-binding enzyme	PF00501	53	C2 domain	PF00168	51	Major Facilitator	PF07690	56

						Superfamily		
Actin	PF00022	51	Chitin binding Peritrophin-A domain	PF01607	51	Myosin head (motor domain)	PF00063	54
Insect cuticle protein	PF00379	51	Helicase conserved C-terminal domain	PF00271	51	Transposase (partial DDE domain)	PF01359	53
C2 domain	PF00168	46	AMP-binding enzyme	PF00501	50	DEAD/DEAH box helicase	PF00270	51
TPR repeat	PF13414	46						
